# Growth of mountain belts in central Asia triggers a new collision zone in central India

**DOI:** 10.1038/s41598-018-29105-2

**Published:** 2018-07-16

**Authors:** I. Koulakov, T. Gerya, B. K. Rastogi, A. Jakovlev, I. Medved, J. R. Kayal, S. El Khrepy, N. Al-Arifi

**Affiliations:** 1 Trofimuk Institute of Petroleum Geology and Geophysics, SB RAS, Prospekt Koptyuga, 3, Novosibirsk, 630090 Russia; 2 Novosibirsk State University, Novosibirsk, Russia, Pirogova 2, Novosibirsk, 630090 Russia; 3 ETH Zurich, Department Of Earth Sciences, Sonneggstrasse 5, Zurich, 8092 Switzerland; 4 Institute of Seismological Research, Gandhinagar, 382009 India; 5 King Saud University, Riyadh, Saudi Arabia, P.O. Box 2455, Riyadh, 11451 Saudi Arabia; 6 National Research Institute of Astronomy and Geophysics, Seismology Department, NRIAG, Helwan, 11421 Egypt

## Abstract

Several unusual strong earthquakes occurred in central India along the Narmada-Son Lineament (NSL) zone, far from active plate boundaries. To understand the role of collisional processes in the origin of this seismicity, we develop a numerical thermomechanical model of shortening between the Indian Plate and Asia. We show that at the final stage of collision, the shortening rate of the high mountain areas slows. The continuing convergence of India and Asia triggers the initiation of a new collision zone in continental part of India. Various geological and geophysical observations indicate that the NSL is a weakest zone with northward thrusting of the thinner central Indian lithosphere underneath the thicker northern part of the Indian Plate. We hypothesize that the NSL was reactivated during the final stage of the India Asia convergence and it will possibly form a new mountain belt within the Indian continent.

## Introduction

**Figure 1:**
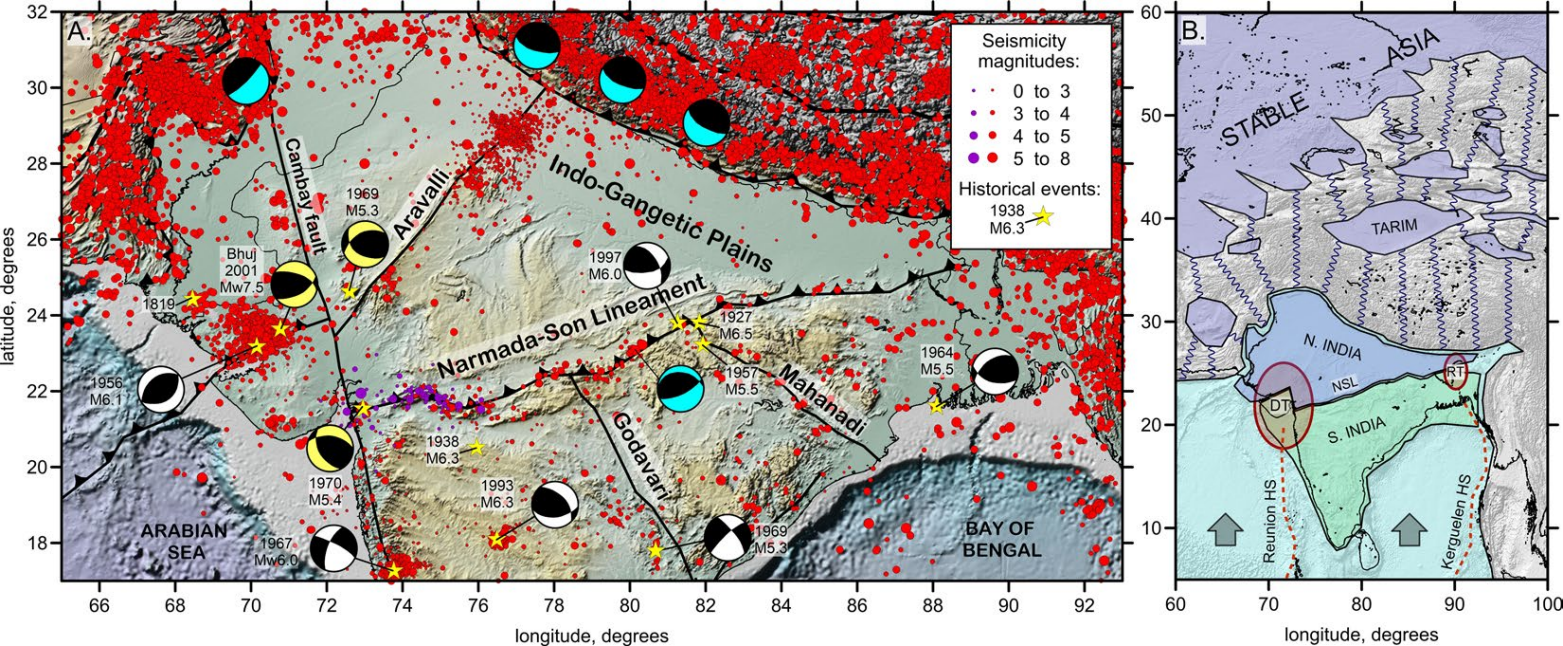
Structural elements in the study region and surroundings. (**A)** Seismicity distributions and major structural elements of the northern part of the Indian Plate. The background is the topography/bathymetry map extracted from Global Multi-Resolution Topography, GMRT^53^. Red circles represent the seismicity from the merged regional (National Centre for Seismology, India) and ISC catalogues, whereas the dark violet circles represent the seismicity recorded by regional stations in the western part of the NSL. Major earthquakes are highlighted with yellow stars; if available, magnitudes and focal mechanisms are presented. Major fault zones, including the NSL, are shown. (**B**) Simplified representation of the “String-and-Block” concept for the description of the India-Asia collision. Blue “strings” represent the zones of thickened crust affected by gravitational spreading. Violet areas represent rigid lithosphere blocks. Light blue represents the incoming Indian Plate. The NSL divides India into two continental blocks: thicker northern (blue) and thinner southern (green) blocks. DT and RT represent the Deccan and Rajamahal traps, respectively; ellipses represent the zones of degraded lithosphere owing to the passage of the corresponding plumes. Red dotted lines represent the tracks of the Reunion and Kerguelen hot spots.

Most of the seismic activity in Asia occurs in highly deformed orogenic belts and is triggered by active deformations of the crust owing to India-Asia collision. However, unusual seismic activity occurs inside the continental Indian Plate (Fig. 1), which is often assumed to be a monolithic unyielding indenter. Although total number of events in the inner parts of the Indian Plate is less than that in the plate boundary zones, such as the Himalayas (Fig. 1), the occurrence of strong earthquakes with magnitudes exceeding Mw 6.0 renders these intraplate regions one of the most hazardous areas in India^1^. For example, the 26 January, 2001 Bhuj earthquake Mw 7.5 occurred in the state of Gujarat in western India far from any tectonic boundaries and killed some 20,000 people^2^. In central India, the May 22, 1997 Jabalpur earthquake Mw 6.0 damaged thousands of houses and killed 39 persons. Among the recent earthquakes in the continental part of India, there are the Killari earthquake (Mw 6.3) in central India in 1993, the Bhadrachalam earthquake (Mw 5.4) in 1967, the Mt. Abu earthquake (Mw 5.3) in the Cambay rift zone in 1969, and the Ongole earthquake (Mw 5.3) in the eastern part of India in 1969^3^ (Fig. 1A).

As shown in Fig. 1A, many strong and moderate earthquakes in the inner parts of the Indian Plate occur along the Narmada-Son Lineament (NSL), which is one of the most prominent tectonic elements in continental India. The NSL is a structure with a length of approximately 1000 km and width of 50 km clearly visible in the topography (Fig. 1A). It is considered as a suture zone between the cratonic blocks that accreted in the geological past starting from 1–1.5 Ga^4^. This long E-W trending system appears to be playing a major role in the present day tectonics and crustal shortening in the peninsular India region. The active contractional deformations at NSL were proposed as early as 1971^5^. There are several observations that suggest that the NSL, a failed rift zone under tensional regime in the geological past, is presently reactivating under a compressional regime^4–6^. Several seismic^7,8^ and magnetotelluric surveys^9,10^ previously performed in different segments of the NSL reveal northward dipping structures that may support the model of underthrusting and shortening in these areas. The focal mechanisms of the main earthquakes along the NSL also indicate compression regime^3,11,12^. Finally, the existing GPS observations directly show that the major contractional deformations of continental India are accommodated in the NSL zone^13–15^. For example, four years measurements at two GPS stations south of Narmada rift at Burwani in the state of Madhya Pradesh (74.90 N 22.04 E) and Sagbara in the state of Maharashtra (73.79 N 21.54 E) indicate deformation rate of more than 3 mm/yr^15^.

The cause of this unusual tectonic activity in intracontinental parts of India is still under debates. To understand the role of collisional processes in the origin of the recent contractional deformations at the NSL, we create a numerical thermomechanical model of shortening between the Indian Plate and Asia. We present also a new regional tomography model giving the information about the lithosphere structure of India that is used to define boundary conditions in the numerical modeling.

## Results

### Lithosphere structure

The information about the lithospheric structure may help to understand the cause of unusual tectonic activity in the intracontinental parts of India. There have been many studies that estimated the lithosphere thickness of India based on different geophysical investigations; however, many of them appear to be inconsistent, and in some cases contradictory. For example, various receiver functions studies^16–18^ report generally unchanged lithosphere thickness at around 100 km in different parts of continental India. An integral study for the whole Indian continent^19^ based on the S-p receiver functions shows significant variations of the lithosphere thickness: relatively thin lithosphere (80–100 km) in the northwestern and southern paths of India and thicker lithosphere (120–140 km) beneath the northeastern part of India. Surface-wave dispersion estimates^20^ appear to be not consistent with another regional^21^ and global studies^22,23^ based on the surface wave tomography that report thick lithosphere of up to 200 km in the northern India and thinner lithosphere (100–150 km) in the Southern India. The later models appear to be consistent with body-wave tomography results^24,25^. These examples show that the problem of the lithosphere thickness determination beneath India is still open and needs further investigations.

**Figure 2:**
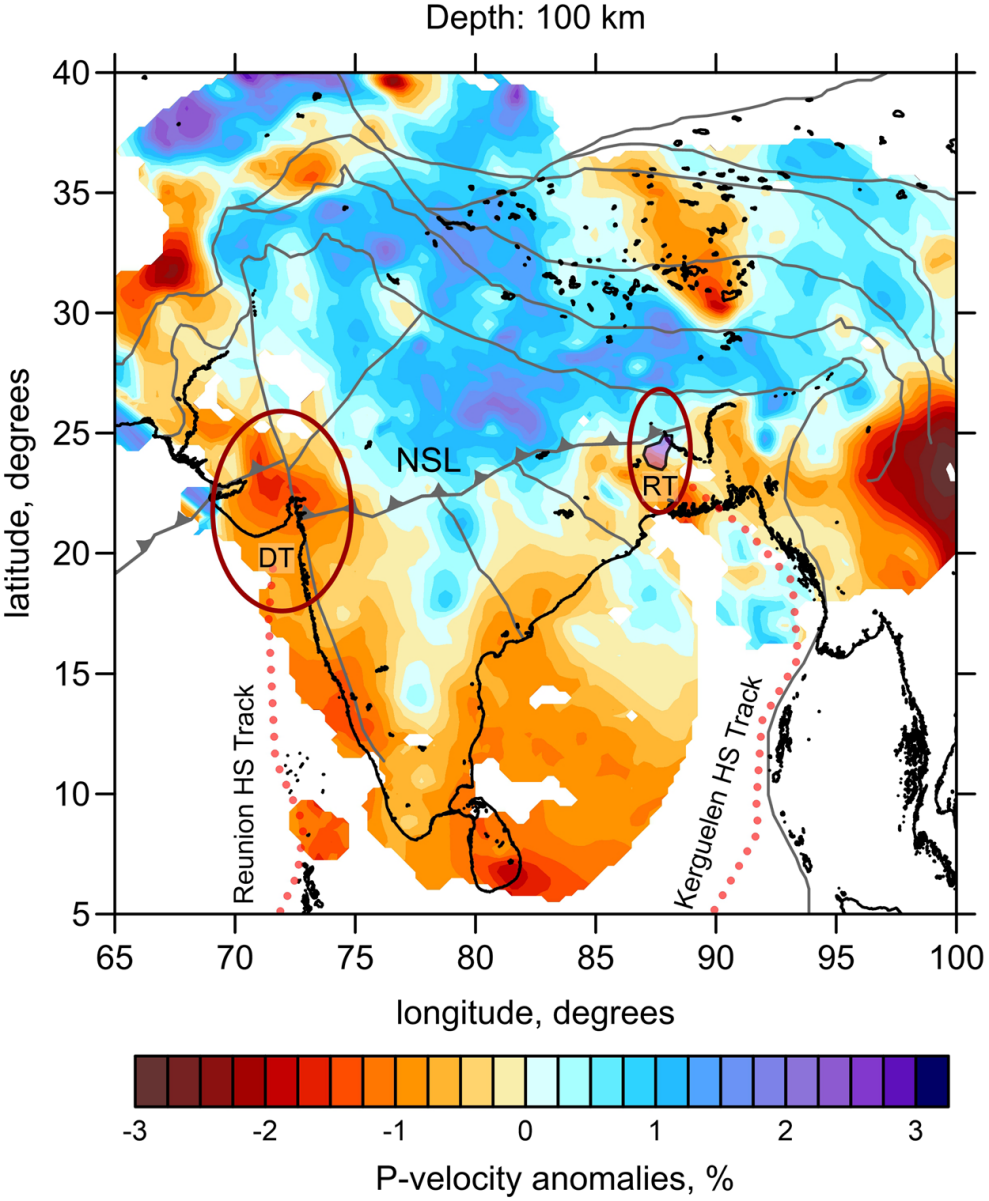
Anomalies of P-velocity derived from regional tomography inversion at 100 km depth representing the lithosphere structure. The line with arrows represents the NSL, and solid gray lines represent other major tectonic boundaries and faults in continental India. Ellipses represent the Deccan (DT) and Rajamahal (RT) traps. Red dotted lines represent the Reunion and Kerguelen hot spot tracks.

Here, we present another seismic model of the upper mantle based on P-wave travel time tomography. The details of the data description, methodology and testing are presented in the Method Section. The results of tomographic inversion are shown as *P*-wave velocity (*Vp*) anomalies at 100 km depth (Fig. 2). The other vertical and horizontal sections of this model are given in Supplementary Information.

In the tomography results, we clearly observe a high *Vp* anomaly beneath the northern part of India extending down to 200 km, which can be interpreted as a thicker continental lithosphere. Beneath southern India, however, the high *Vp* anomaly is less prominent and alternates with lower *Vp* anomalies. Based on this, as well as previous geophysical studies, we propose that the NSL delimits thicker lithosphere in northern India and thinner lithosphere in southern India. Such a step-shaped structure may be the most plausible place for the initiation of underthrusting of the thinner plate underneath the thicker one, as shown by previous modeling results^26^.

At the western margin of India at the depth of 100 km (Fig. 2), we observe a low-velocity anomaly with the strongest amplitude beneath the Deccan traps. We propose that this anomaly represents a zone of the thinned lithosphere degraded by the Reunion-Deccan hot spot. Previous numerical models^27^ have shown that trap-generating plumes may quickly destroy the continental lithosphere owing to the magmatism-induced lithospheric weakening and foundering. In the eastern part of India, we observe another low-velocity anomaly that may be associated with the Rajamahal traps. Weakening of the lithosphere owing to the presence of these two features at both ends of the NSL might be another factor facilitating the origin of a regional-scale fracture zone in the lithosphere.

### Thermomechanical model of collision and discussion

Here, we present a geodynamic scenario accounting for the unusual seismicity in the intracontinental areas of India. The collision of the Indian Plate with Asia has led to the origin of the largest mountain belt on the Earth between the Indian Plate and stable Asia containing Siberian, West Siberian, Kazakhstan, and other plates^28^. Most of the shortening is accommodated in the folded belts of weaker lithosphere squeezed between several rigid terranes as schematically shown in Fig. 1B. The shortening leads to mountain building and gradual crustal thickening. This causes the growth of topographic loads and creates outward-directed forces from orogenic areas, which in turn increases the compressional stresses in the interior of the colliding plates. Consequently, lithospheric shortening migrates from already thickened (initially weak) lithospheric sections to new (initially stronger) lithospheric domains. In the case of the India-Asia collision, such subsequent shortening occurred in a series of orogenic belts from the Himalayas and Tibet to Tien Shan, Altay and Sayan^29^. We propose that, after achieving a critical compression in the orogenic belts of Asia, the shortening may jump to the continental Indian Plate and become focused at the NSL.

**Figure 3:**
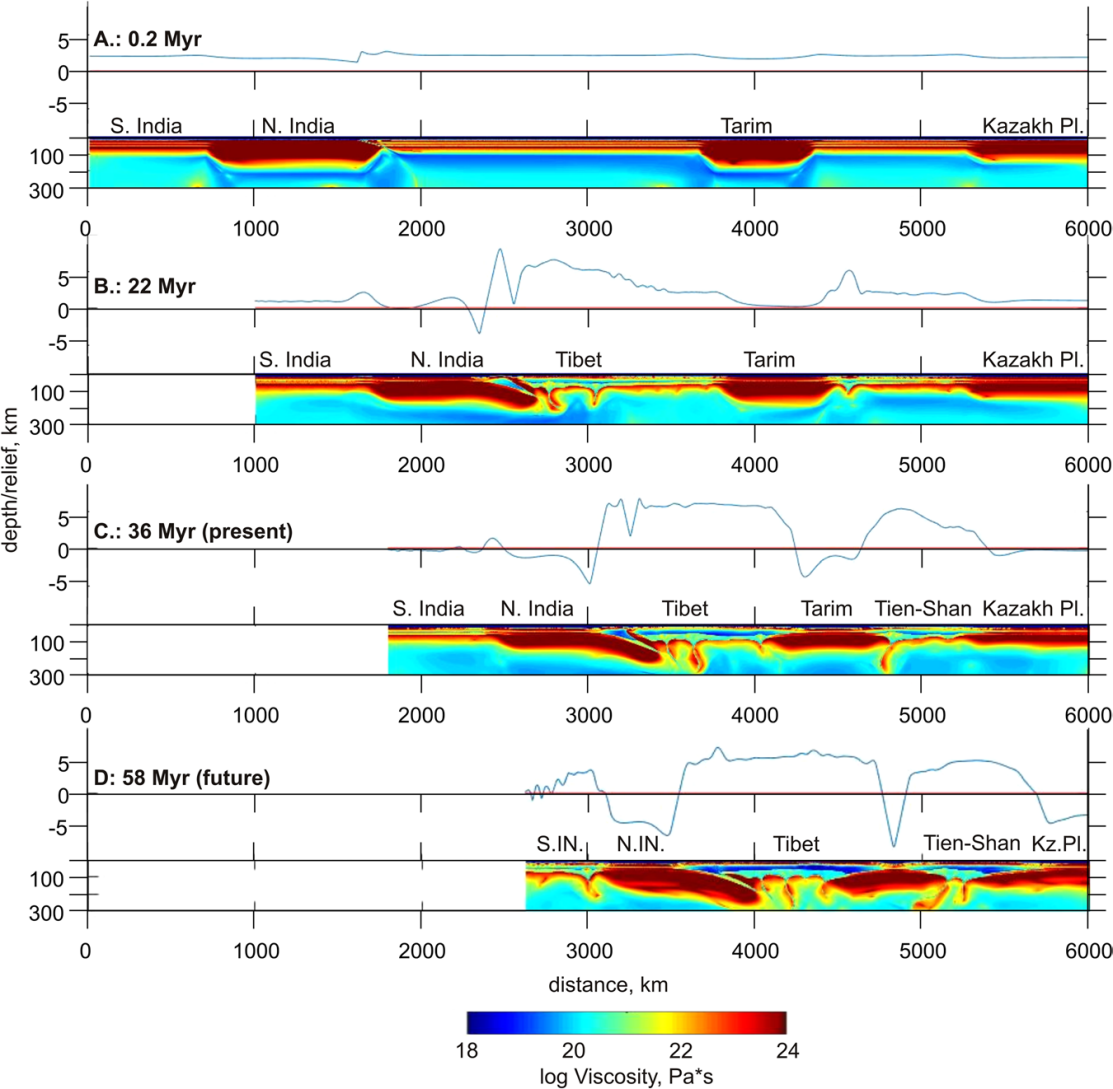
Results of numerical modeling of subsequent mountain building owing to shortening during the collision between India and stable Asia. The evolution of the topography and viscosity distributions in the uppermost mantle during four episodes of collision is shown. S.IN and N.IN indicate the southern and northern parts of the Indian continent, respectively.

In order to simulate the evolution of the lithospheric shortening owing to the India-Asia collision, we have created a simplified two-dimensional numerical visco-elasto-plastic thermomechanical model (Fig. 3A). It contains several continental lithospheric units corresponding to major regions identified within the Indian and Asian plates and differing in the initial thickness (and thus strength) of the continental lithosphere (details of the numerical approach, initial setup, and parameter study for the model are presented in the Method Section). The India-Asia-like collision in this model is initiated by prescribing an inclined northward dipping lithospheric-scale weak zone corresponding to the terminated subduction suture along which the Tethys Ocean subducted. The results of numerical experiments systematically show the migration of deformation from initially weaker (i.e., thinner) to initially stronger (i.e., thicker) lithospheric sections, which is associated with the gradual growth of intra-plate compressional stresses in both the Indian and Asian model domains.

Figure 3 shows the evolution of the reference experiment, which reproduces the first-order dynamics of the India-Asia collision. During the first 20 Myr, most of the mountain building occurred in the Himalayas and Tibet (Fig. 3B). However, after reaching some critical shortening between India and Tarim, a new mountain system started to form between the Tarim and Kazakh Plates (Fig. 3C). This appears to be consistent with previous geological studies^30^ that assumed a delay of approximately 30 Myr of the Tien Shan orogenesis with respect to the beginning of the Indian collision. In our model, we observe that the mountains of Tien Shan gradually grow from south to north, as identified from geological observations^31^. The model in Fig. 3C roughly represents the state of the collision belts in our time.

We have continued the calculations to forecast the development of the collision in the future (Fig. 3D). After some time, the shortening of central Asia becomes inefficient; the thickened crust in the mountain areas starts to collapse gravitationally, thus imposing strong outward-directed forces preventing further shortening^32,33^. At this stage, a new collision zone nucleates in central India at the boundary between the thicker northern and thinner southern lithospheric domains identified within the Indian continent, which corresponds to the NSL. The numerical model predicts that, at the forthcoming stages of the collision, the NSL will tend to develop into a northward dipping subduction of the thinner southern part of the Indian Plate, and a new intracontinental continental collision zone will form. At the same time, we admit that the real collisional processes within the continental Asia are more complicated than presumed by our simplified modeling setup. Indeed, besides the seismicity along the NSL, we observe numerous earthquakes along other fault zones, such as Aravalli, Godavai and Mahanadi (Fig. 1A). This implies that the shortening of the Indian continent will possibly occur along several branches. However, we claim that the NSL will play the major role in breaking the Indian continent due to inherited structural features of the lithosphere.

Thus, the geological-geophysical observations and numerical modeling results indicate that the NSL is the most plausible candidate for breaking the lithosphere and forming a new collision zone to accommodate shortening owing to the India-Asia collision. It is possible that the observed topography high along the NSL represents the initiation of a new mountain chain that will separate the stable terrain of northern India (similar to that of Tarim) from the south. The major shortening of the India-Asia collision will be subsequently accommodated along this region owing to the underthrusting of the southern part of the Indian plate underneath the NSL.

## Methods

### Data and algorithms for tomography

We used the regional tomography algorithm^34^ with global travel time data from the International Seismological Centre^35^ corresponding to the period 1964–2014. For the selected region, we consider any data corresponding to the ray paths passing through the study volume. This includes rays from earthquakes located in the study area recorded by stations worldwide, and those from teleseismic events recorded by stations located in the study area (Fig. S1A and B). Prior to their use in tomography, the data were reprocessed; this included relocating sources and rejecting outliers^36^. To locate events, we used the one-dimensional velocity model AK135^37^.

This area has previously been part of another model calculated using the same algorithm^25^. However, the previous study covered only the northern part of India. Further, our study includes data from 2005–2014, which were not available in the previous study. Ten years of additional records have provided significant amount of data, especially those corresponding to new stations in India that drastically improved the ray coverage.

The inversion was performed separately in a series of overlapping areas covering the entire study region. We used three regions, each with a radius of 8° (Supplementary Materials, Fig. S1). We defined the depth of the study volume at 1,000 km; however, we mostly considered results down to a depth of 700 km because deeper structures might have been affected by anomalies located outside the study area. The parameterization of velocity distribution was performed using a set of nodes distributed on horizontal levels at depths of 25, 50, 75, 100, 150, 220, 290, 360, 430, 500, 570, 640, 710, 800, and 900 km. At each depth level, the nodes were distributed according to the density of rays; denser ray coverage corresponded to smaller node spacing. The minimum spacing was set at 30 km. To avoid artifacts related to grid geometry, we performed the calculations for two different grids with basic orientations of 0 and 45° and then averaged the results.

The inversion was performed simultaneously for the P and S velocity anomalies and for source corrections. When data from the events located in the study area were used, we considered four unknown parameters corresponding to shifts of sources in space and time. For the teleseismic data, we inverted for one parameter per event to represent the uncertainty of time determination outside the study volume. The matrix was inverted using the LSQR method^38,39^. The stability of the inversion was controlled using additional equations determining the amplitude and flattening of the resulting velocity anomalies. The values of the damping coefficients were set according to several trials with synthetic models.

The results of tomographic inversion are shown in the main paper in one horizontal section at 100 km depth (Fig. 2) and in Supplementary Materials in two horizontal sections at 300 and 500 km depth (Fig. S2) and two vertical sections here (Fig. S3). Here, we present the results for the P-wave velocity anomalies only, because the S-data are almost one tenth of the P-data, and the resulting S-model does not appear sufficiently stable.

In Supplementary Materials in Fig. S4, we present the results of the checkerboard test, giving the information about the spatial resolution for the retrieved models. The synthetic model consisted of alternate positive and negative anomalies with a magnitude of 3% and lateral size of 5° × 5° km. With increasing depths, the anomalies changed signs at 200, 400, and 600 km. The synthetic data were computed along the same ray paths to derive the experimental data model, and these were perturbed by random noise with an average deviation of 0.5 s. The periodic checkerboard anomalies were defined throughout the Earth, whereas the inversion was performed in selected circular regions. This allowed us to explore the effect of anomalies located outside the study area that were taken into account when computing the synthetic data. The results of the checkerboard recovery are shown in Supplementary Materials (Fig. S4). The general locations of all anomalies were reconstructed correctly; however, we observed some diagonal smearing related to the predominant orientations of the ray paths. We found fairly good vertical resolution, allowing us to clearly recover the sign changes with depth.

In addition, we have performed a synthetic test with realistic shapes of anomalies, which are presented in horizontal and vertical sections (Figs S5 and S6). The anomalies are defined within a series of polygonal blocks in some depth intervals. The recovery results show that the lateral configurations of all anomalies are restored correctly. In vertical sections, we see that the anomalies representing the lithosphere of variable thickness are resolved at correct depths. Both these tests support the reliability of the derived results.

Results of the tomographic inversion are shown in three horizontal sections (Fig. 2 of the main paper and Fig. S2) and two vertical sections (Fig. S3). Apart from the results related to the Indian peninsula, the model includes some surrounding areas. At least two structures were consistently retrieved in several previous studies and could therefore be used as a natural benchmark for the present model. One of the brightest patterns in most Asian regional tomography studies is the well studied high Vp structure beneath the Pamir–Hindu Kush, which is associated with the distribution of seismicity at intermediate depths (down to 200 km). Images of this high Vp anomaly were consistently obtained by different authors using different data sets and algorithms^40–42^. The second benchmark structure is an elongated north–south directed high Vp anomaly beneath the Burmese arc marked with the intermediate depth seismicity. In our model, we reveal this anomaly as was reported in previous studies^43–46^. These two examples strongly show that our present model is equally stable for areas that were not covered by previous studies.

### Modeling of continental collision

#### Modeling approach

The numerical thermomechanical visco–elasto–plastic 2D C-code I2ELVIS used to model continental collision is based on a finite difference method, applied on a staggered Eulerian grid, and uses a marker-in-cell technique^47,48^. The momentum, mass, and energy conservation equations are solved on the Eulerian grid, and physical properties are transported by Lagrangian markers that move according to the velocity field interpolated from the grid. Non-Newtonian visco–elasto–plastic rheology based on experimentally calibrated flow laws is used in the model (Supplementary Materials, Table S1). The full details of this method, that allow its reproduction, are provided elsewhere^47,48^.

*Numerical model design*. The initial model setup (Supplementary Materials, Fig. S5) is 6,000 km wide, 300 km deep, and resolved with a regular rectangular grid of 601 × 151 to 1,201 × 151 nodes (varied in different experiments, Table S2) containing 1.8 million randomly distributed Lagrangian markers. The upper and right-hand boundaries of the model have free slip mechanical boundary conditions. A constant convergence velocity of 4.7 cm/year is prescribed at the left-hand boundary. The downward lower boundary velocity was defined by the volume conservation condition of the computational domain, and was thus shortened and thickened at every time step. The free surface boundary condition above the crust is implemented using a 20-km-thick “sticky” air layer^49,50^ with low density (1 kg/m^3^) and viscosity (10^18^ Pa-s). The initial thermal and lithological structure of the model (Fig. S5) was defined by prescribing several continental lithospheric units corresponding to major regions identified within the Indian and Eurasian plates and differing in the initial thermal gradient of the continental lithosphere (Fig. S5). In simple terms, initially uniform 40-km-thick continental crust is composed of 15 km felsic upper crust, 10 km intermediate middle crust, and 15 km mafic lower crust (layers thickness varied in different experiments, Table S2). The used initially uniform crustal structure is simplified and neglects, for example, lateral heterogeneity of crustal thickness of the Indian continent^51,52^. This simplification is mainly due to the large uncertainties of our knowledge of initial crustal thickness for different continental lithospheric units. This thickness will likely have an opposite effect compared to the initial thickness of the lithosphere due to the rheological weakness of the crust compared to the lithospheric mantle (Table S1). The initial crustal thickness evolves strongly during numerical experiments, in which crust predominantly thickens in regions of initially thin and thus warm and weak lithosphere (Table S2). A rightward inclined lithospheric-scale weak zone (a terminated subduction suture of the Tethys Ocean) marks the initiation site of an India–Eurasia-like collision. Simplified linear geothermal gradients were used in different lithospheric sections (thicknesses varied in different experiments, Table S2) at 273 K (at the surface) and 1,573 K (mantle potential temperature). An adiabatic thermal gradient of 0.5 K/km was initially prescribed in the asthenospheric mantle. Temperature-dependent thermal conductivity was used for the mantle and crust (Table S1). The thermal boundary conditions are 273 K (upper), 1,713 K (lower), and zero heat fluxes on the left- and right-hand boundaries. To insure efficient heat transfer from the surface of the crust, the temperature of the “sticky” air/water is kept constant at 273 K. A gravitational acceleration of 9.81 m/s^2^ was used for the model. It should be noted that the 2D model used in our study neglects lateral variability of 3D deformation pattern of India-Asia collision zone. However we think that this simplified model is sufficient for the purpose of our study focused on the transfer of deformation with time from initially weaker to initially stronger lithospheric regions.

#### Visco–elasto–plastic rheological model

The viscous, elastic, and brittle (plastic) properties (Table S1) were implemented via evaluation of the effective viscosity of the material. For the ductile materials, the contributions from different flow laws such as dislocation and diffusion creep were considered via the computation of the inverse average ductile viscosity *η*_ductile_

1 $$\frac{1}{{\eta }_{{\rm{ductile}}}}=\frac{1}{{\eta }_{{\rm{newt}}}}+\frac{1}{{\eta }_{{\rm{powl}}}}$$

where *η*_*newt*_ and *η*_*powl*_ are effective viscosities for diffusion and dislocation creep, respectively, computed as

2 $${\eta }_{{\rm{newt}}}=\frac{{A}_{D}}{2{\sigma }_{{\rm{cr}}}^{n-1}}\exp (\frac{E+PV}{RT}),$$

3 $${\eta }_{{\rm{powl}}}=\frac{1}{2}{A}_{D}^{\frac{1}{n}}\exp (\frac{E+pv}{nRT}){\dot{\varepsilon }}_{II}^{\frac{1}{n}-1},$$

where *P* is the pressure, *T* is the temperature (in K), ${\dot{\varepsilon }}_{II}=\sqrt{1/2{({\dot{\varepsilon }}_{ij})}^{2}}$ is the second invariant of the strain rate tensor, *σ*_cr_ is the diffusion–dislocation transition stress^36^, and *A*_*D*_, *E*, *V*, and *n* are experimentally determined flow law parameters (Table S1), which denote the material constant, activation energy, activation volume, and stress exponent, respectively.

The ductile rheology is combined with a brittle (plastic) rheology to yield an effective viscous-plastic rheology by using the following upper limit for the ductile viscosity:

4 $${\eta }_{ductile}\le \frac{C+\varphi P}{2{\dot{\varepsilon }}_{II}},$$

where *P is* the pressure, *ϕ* is the internal friction coefficient (Table S1), and *C* is the tensile rock strength at *P* = 0 (Table S1). Elasticity is implemented based on the incompressible visco–elasto–plastic Maxwell model^47,48^. The shear moduli (μ) of different materials are specified in Table S1.

#### Numerical results

We performed 12 numerical experiments, varying the crustal layering and the initial lengths and lithospheric thicknesses of different model sections (Table S2). The numerical results show systematic migration of deformations from initially weaker (i.e., thinner) to initially stronger (i.e., thicker) lithospheric sections associated with the gradual growth of intra-plate compressional stresses in both the Indian and Eurasian model domains. This general tendency is not affected by the variations of initial model geometry, which have been explored; it only influences deformation dynamics in the weakest (Tibet, Tien–Shan) lithospheric sections (Table S2).

## Electronic supplementary material

Supplementary information
