# Supplementary material for: Growth of mountain belts in central Asia triggers a new collision zone in central India
Source: Sci Rep. 2018 Jul 16;8:10710. doi: 10.1038/s41598-018-29105-2 (PMC6048038; doi:10.1038/s41598-018-29105-2)
Supplement: Supplementary file 1 — Supplementary information [file 41598_2018_29105_MOESM1_ESM.docx]

**Growth of mountain belts in central Asia triggers a new collision zone in central India**

I. Koulakov, T. Gerya, B.K. Rastogi, A. Jakovlev, I. Medved, J.R. Kayal., S. El Khrepy, and N. Al-Arifi

**Supplementary Information** includes 7 additional figures supporting the main results of the paper:

**Figure S1.** Distributions of data. **A.** Stations (light blue triangles) and earthquakes (light red dots) from the International Seismological Centre catalogues used in this study. The green circles indicate areas where the independent inversions were performed. Dark blue triangles depict seismic stations outside the study area that recorded events in the study area. Dark red dots are earthquakes that occurred outside the study area but were recorded by stations in the study area. Grey lines depict the Narmada–Son Lineament and the major faults in continental India. **B.** Distributions of seismic rays in the depth interval from 0 to 200 km depth. Blue, red and black paths correspond to three different circular areas indicated as 1, 2 and 3. Yellow triangles and dots depict seismic stations and events used in this study.

**Figure S2.** Anomalies of P-velocity derived from regional tomography inversion at depths of 300 km and 500 km. The line with arrows represents the NSL, and solid gray lines represent other major tectonic boundaries and faults in continental India. Ellipses represent the Deccan (DT) and Rajamahal (RT) traps. Red dotted lines represent the Reunion and Kerguelen hot spot tracks.

**Figure S3.** P-velocity anomalies derived from regional tomography inversion in one horizontal and two vertical sections. The legend for the horizontal section is same as in Fig. 2 of the main paper. An exaggerated relief (in black) is given above the vertical sections. NSL is Narmada–Son Lineament.

**Figure S4.** Checkerboard test. The shapes of the initial anomalies are highlighted with grey lines. The anomalies have a size of 5 × 5 degrees in lateral directions. With increasing depth, they change signs at 200, 400, and 600 km; thus, the recovery results correspond to the middle depth of each layer. The grey line with arrows depicts the Narmada–Son Lineament.

**Figure S5.** Results of the synthetic test with realistic shapes of P-wave velocity anomalies. Upper row present the synthetic anomalies in different depths. Numbers indicate amplitudes of anomalies. Lower row show the recovery results for the P-wave velocity anomalies at the same depths. Thin contours highlight the true shapes of anomalies at the current depth. The ranges of the color scale for plotting the anomalies are indicated in each map.

**Figure S6.** Same synthetic test as in Figure S5, but with recovered P-wave velocity anomalies in four vertical sections. The locations of the sections are shown in maps in the upper part of the figure. The locations of the synthetic anomalies are indicated with thin contour lines. The ranges of the color scale for plotting the anomalies are indicated in each panel.

**Figure S7.** Initial setup used for the reference model shown in Fig. 3.

A. B.


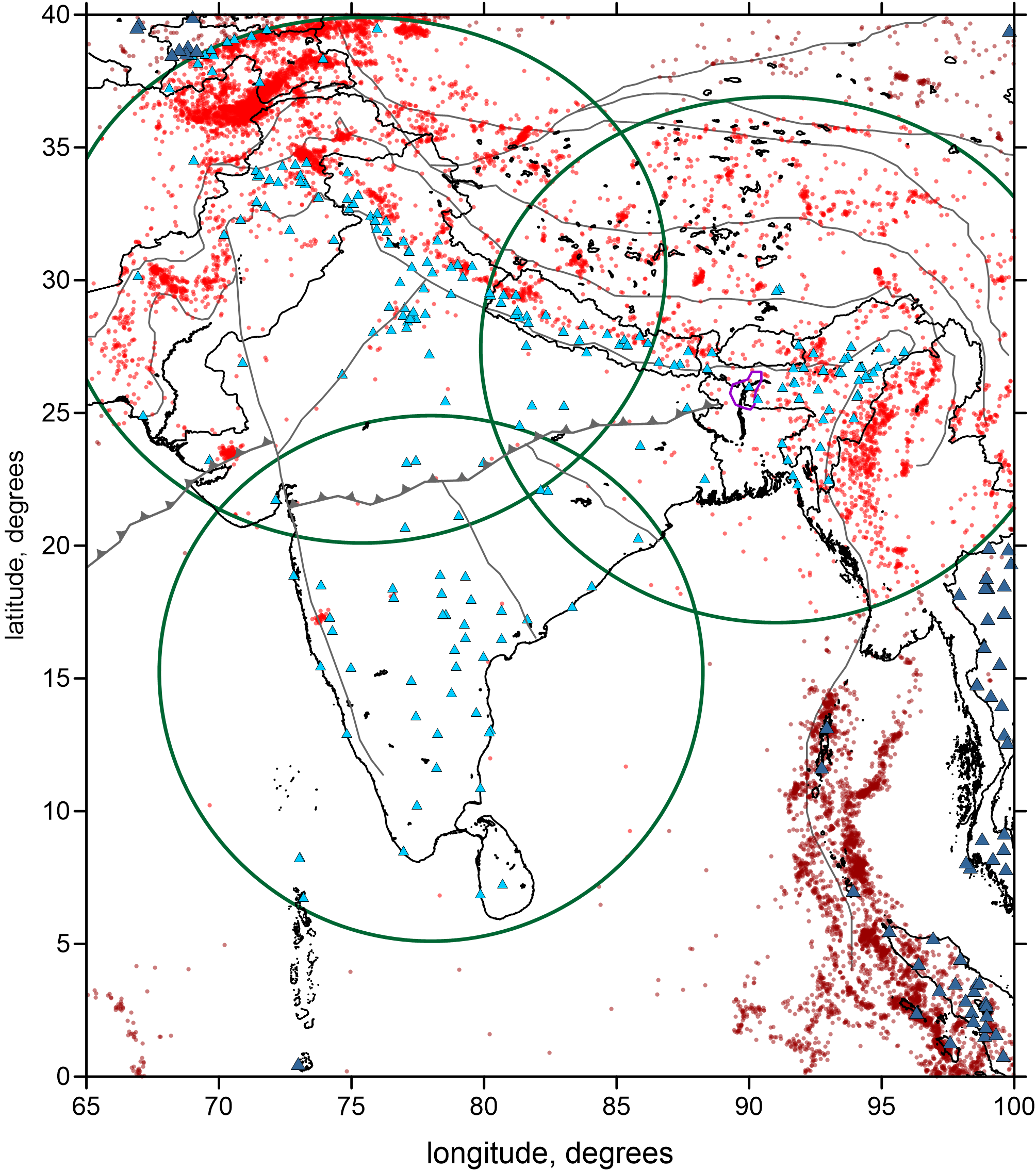

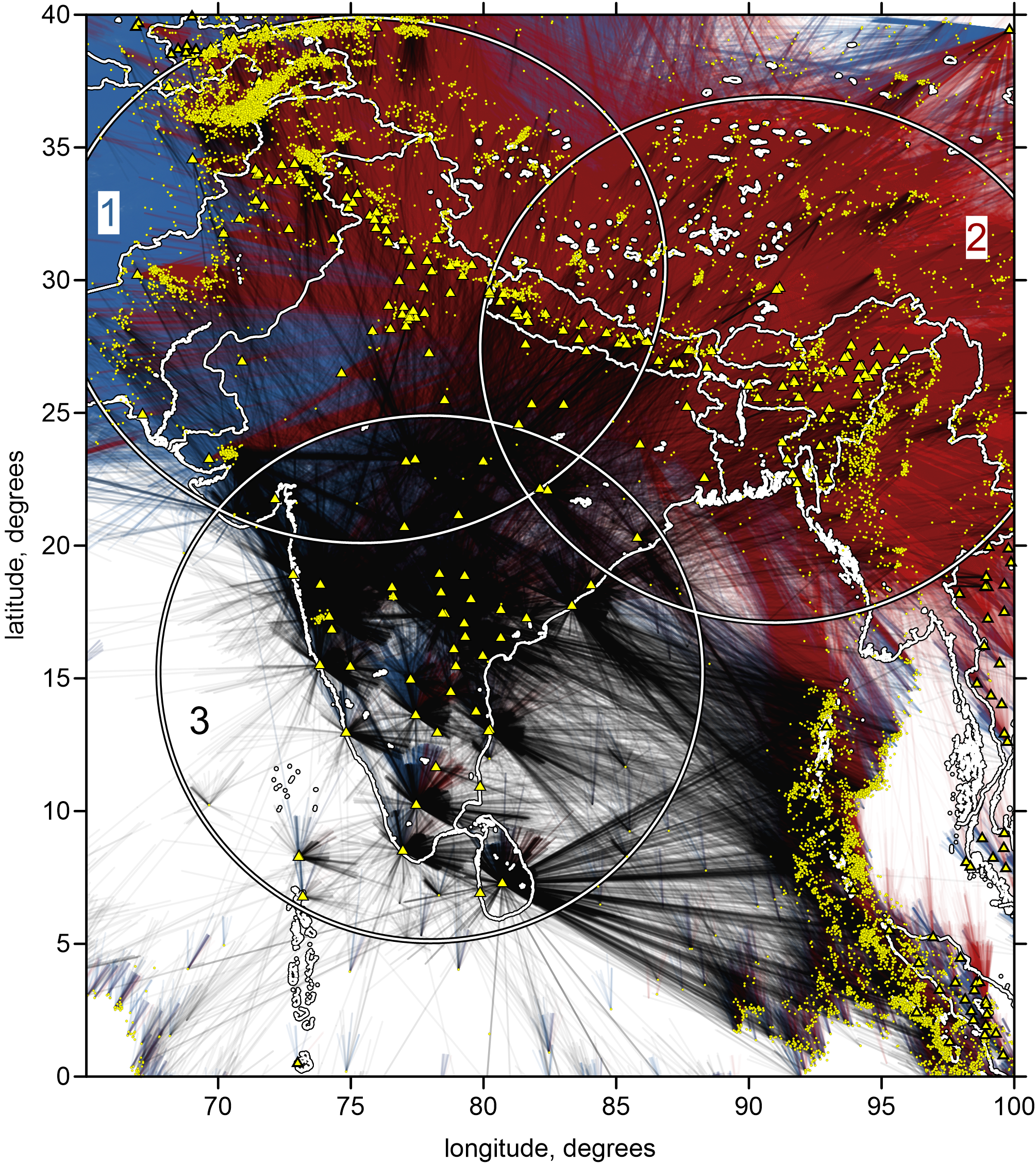


Figure S1. Distributions of data. A. Stations (light blue triangles) and earthquakes (light red dots) from the International Seismological Centre catalogues used in this study. The green circles indicate areas where the independent inversions were performed. Dark blue triangles depict seismic stations outside the study area that recorded events in the study area. Dark red dots are earthquakes that occurred outside the study area but were recorded by stations in the study area. Grey lines depict the Narmada–Son Lineament and the major faults in continental India. B. Distributions of seismic rays in the depth interval from 0 to 200 km depth. Blue, red and black paths correspond to three different circular areas indicated as 1, 2 and 3. Yellow triangles and dots depict seismic stations and events used in this study.


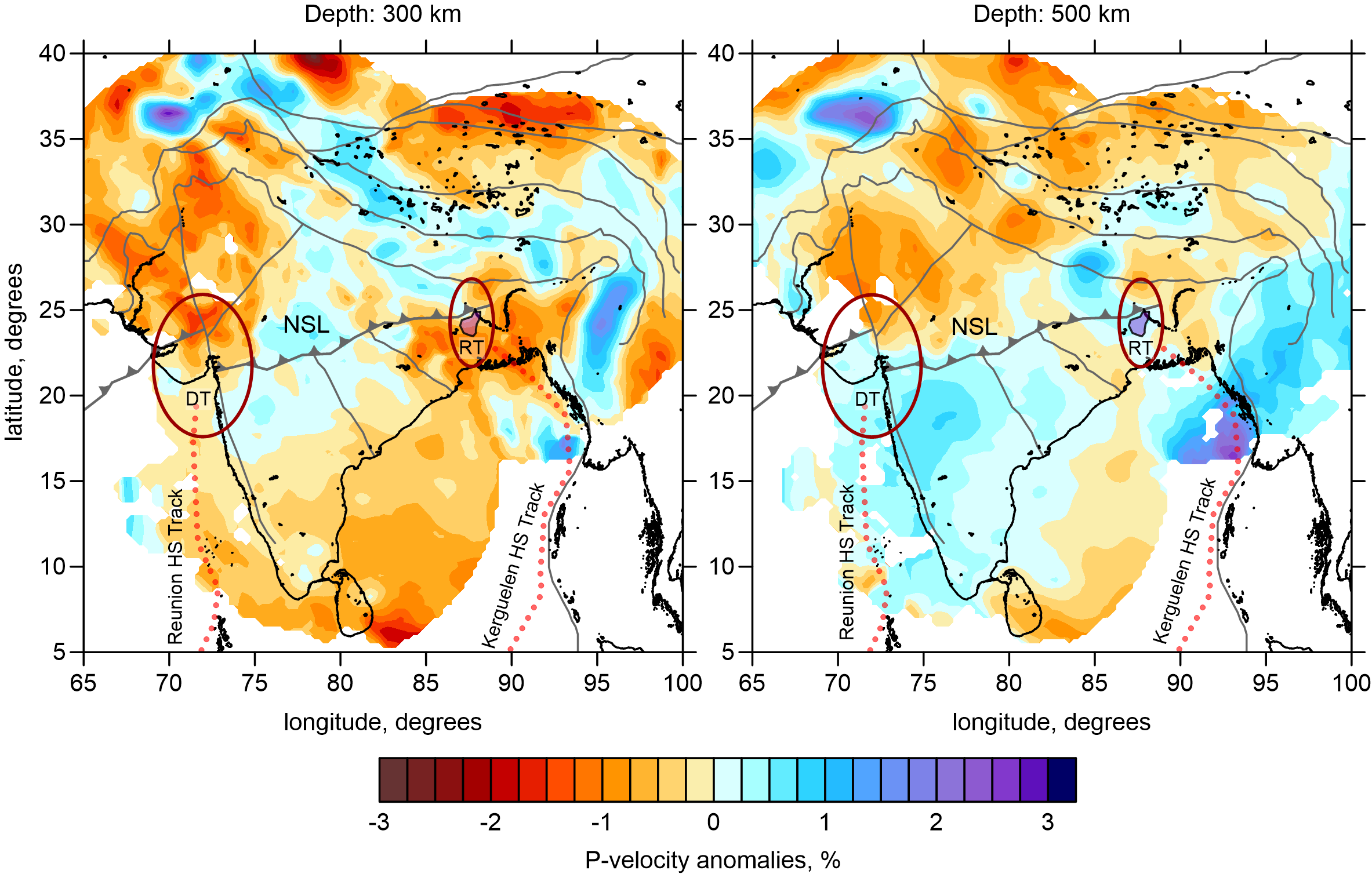


Figure S2. Anomalies of P-velocity derived from regional tomography inversion at depths of 300 km and 500 km. The line with arrows represents the NSL, and solid gray lines represent other major tectonic boundaries and faults in continental India. Ellipses represent the Deccan (DT) and Rajamahal (RT) traps. Red dotted lines represent the Reunion and Kerguelen hot spot tracks.


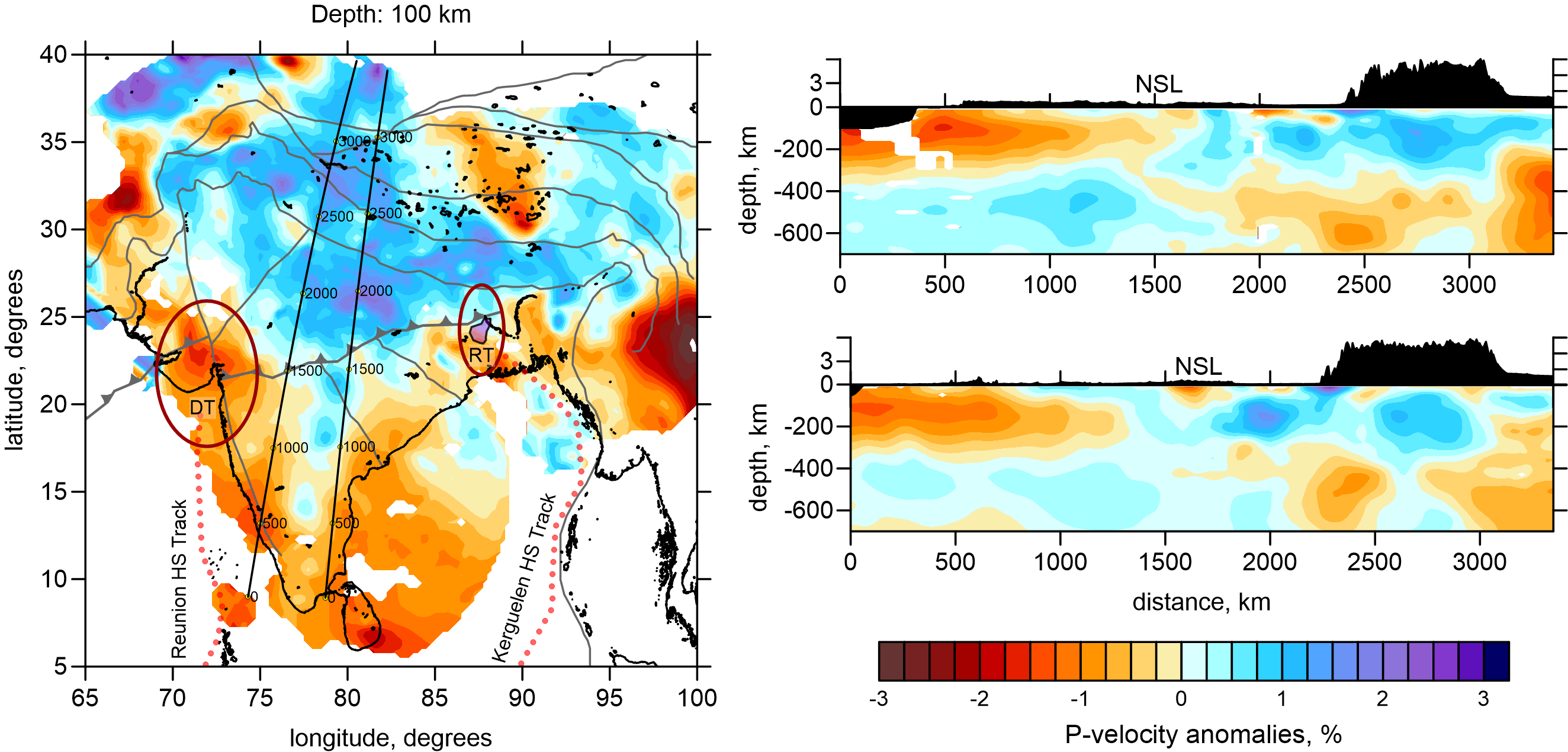


Figure S3. P-velocity anomalies derived from regional tomography inversion in one horizontal and two vertical sections. The legend for the horizontal section is same as in Fig. 2 of the main paper. An exaggerated relief (in black) is given above the vertical sections. NSL is Narmada–Son Lineament.


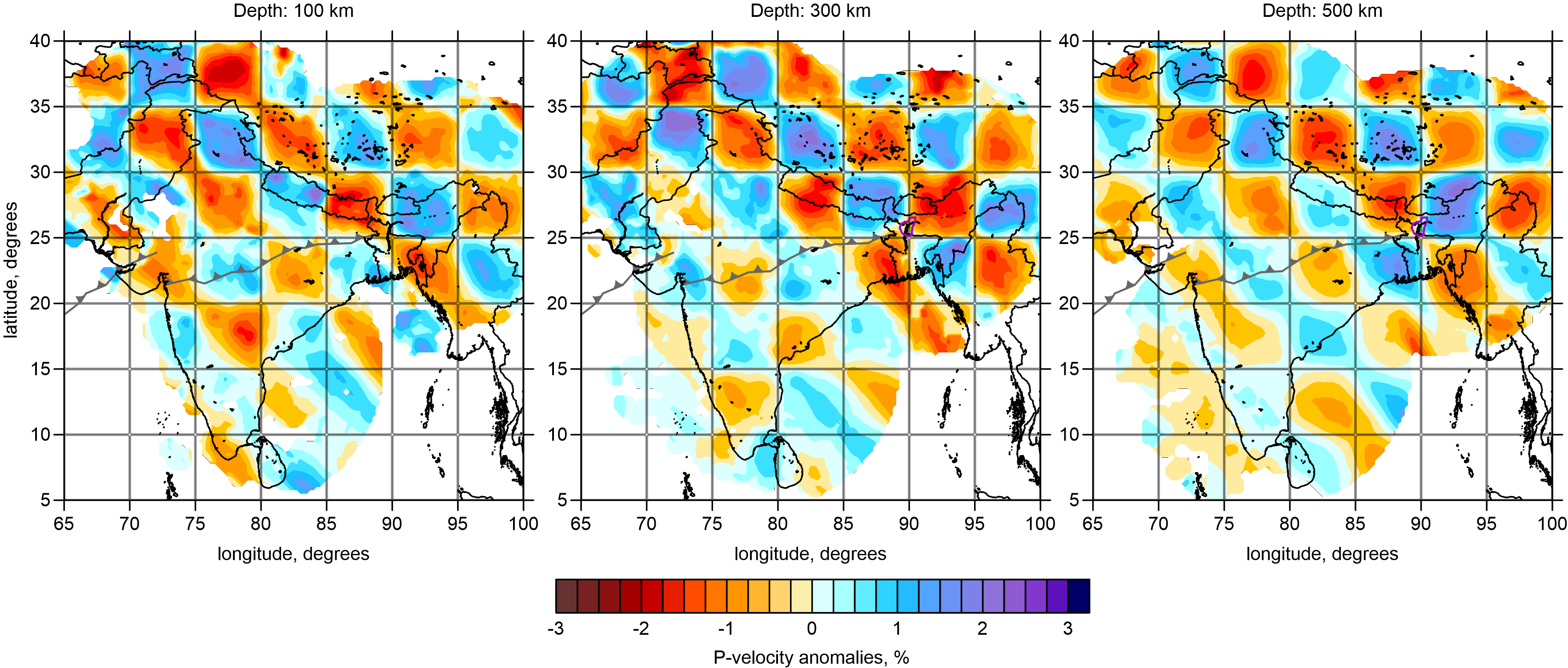


Figure S4. Checkerboard test. The shapes of the initial anomalies are highlighted with grey lines. The anomalies have a size of 5 × 5 degrees in lateral directions. With increasing depth, they change signs at 200, 400, and 600 km; thus, the recovery results correspond to the middle depth of each layer. The grey line with arrows depicts the Narmada–Son Lineament.


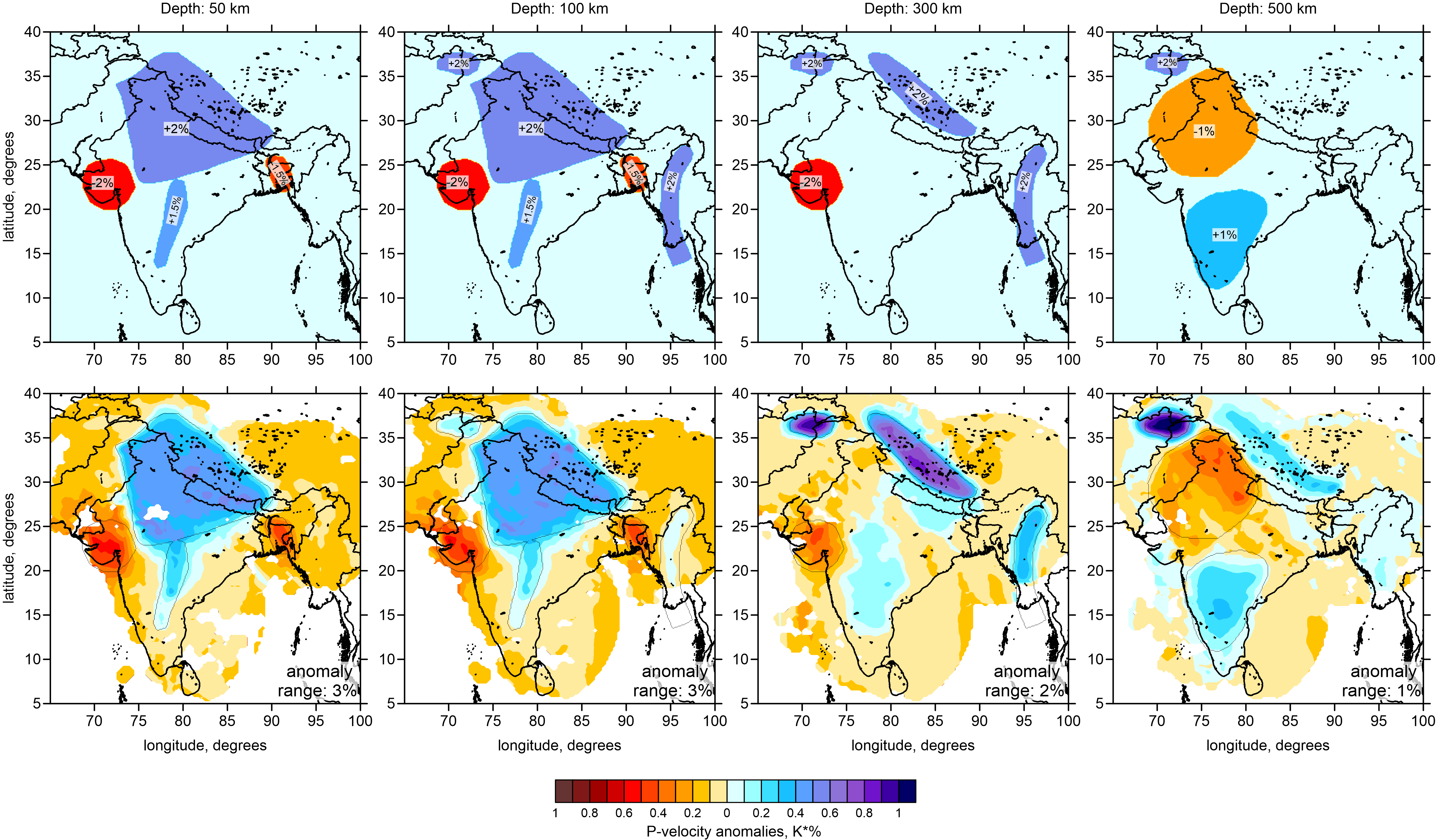


Figure S5. Results of the synthetic test with realistic shapes of P-wave velocity anomalies. Upper row present the synthetic anomalies in different depths. Numbers indicate amplitudes of anomalies. Lower row show the recovery results for the P-wave velocity anomalies at the same depths. Thin contours highlight the true shapes of anomalies at the current depth. The ranges of the color scale for plotting the anomalies are indicated in each map.


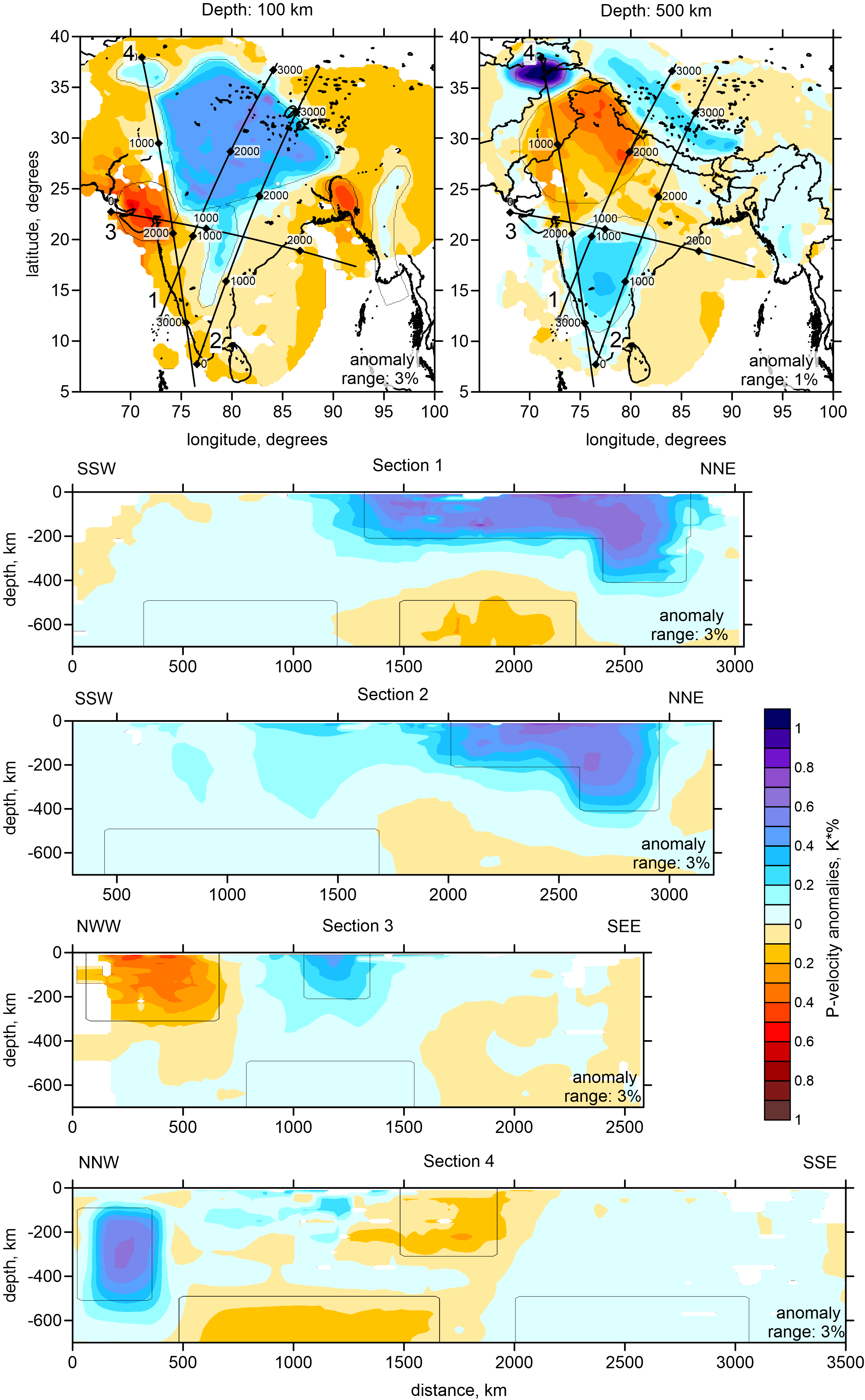


Figure S6. Same synthetic test as in Figure S5, but with recovered P-wave velocity anomalies in four vertical sections. The locations of the sections are shown in maps in the upper part of the figure. The locations of the synthetic anomalies are indicated with thin contour lines. The ranges of the color scale for plotting the anomalies are indicated in each panel.

**
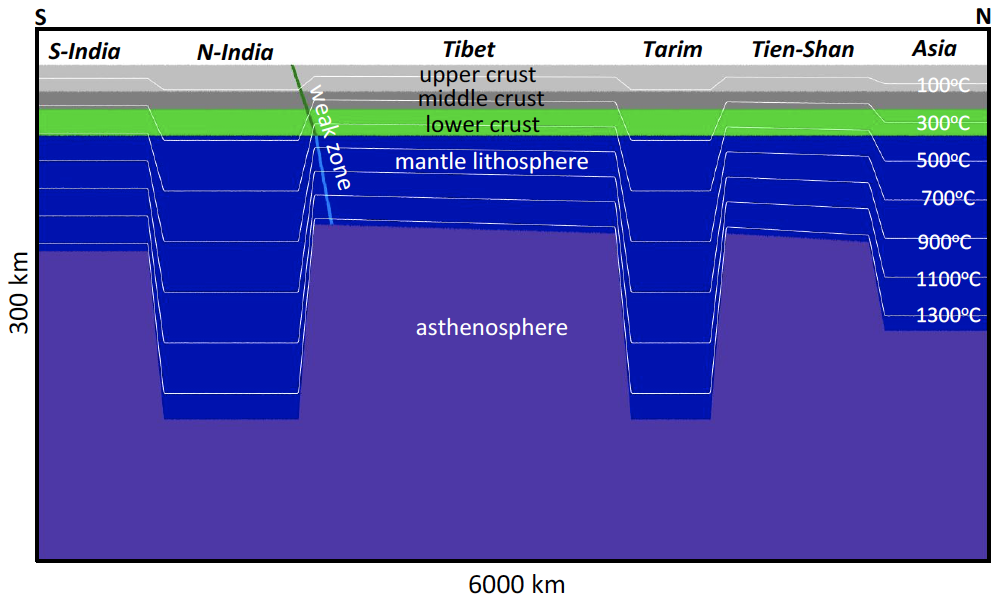
**

Figure S7. Initial setup used for the reference model shown in Fig. 3.

Table S1. Physical properties of rocks used in numerical experiments.

| **Material** | **Density**  **(kg/m^3^)** | **Thermal**  **conductivity,**  **(W/m/K, at *T*_K_)** | **Flow law** |
| --- | --- | --- | --- |
| Upper and middle crust | 2,700 | 0.64+807/(*T*+77) | wet quartzite, *A_D_* =1.97×10^17^ Pa*^n^*s, *n* = 2.3,  *E* = 154000 J/mol, *V* = 0 J/mol/MPa,  σ_cr_ = 3 × 10^4^ Pa, C = 1 MPa, φ = 0.3, μ = 25 GPa |
| Lower crust | 3,000 | 1.18+474/(*T*+77) | Plagioclase, An_75_, *A_D_* = 4.80 × 10^22^ Pa*^n^*s, *n* = 3.2,  *E* =238000 J/mol, *V* = 0 J/mol/MPa,  σ_cr_ = 3 × 10^4^ Pa, C = 1 MPa, φ = 0 .6–0.3^c^, μ = 25 GPa |
| Lithospheric and asthenospheric mantle | 3,300 | 0.73+1293/(*T*+77) | dry olivine, *A_D_* = 3.98×10^16^ Pa*^n^*s, *n* = 3.5,  *E* = 532000 J/mol, *V*= 8 J/mol/MPa,  σ_cr_ = 3 × 10^4^ Pa, C = 1 MPa, φ = 0 .6, μ = 67 GPa |
| Weak zone (crust) | 3,000 | 1.18+474/(*T*+77) | wet quartzite, *A_D_* = 1.97×10^17^ Pa*^n^*s, *n* = 2.3,  *E* = 154000 J/mol, *V*= 0 J/mol/MPa,  σ_cr_ = 3 × 10^4^ Pa, C = 1 MPa, φ = 0.05, μ = 25 GPa |
| Weak zone (mantle) | 3,250 | 0.73+1293/(*T*+77) | wet olivine, *A_D_* = 5.01 × 10^20^ Pa*^n^*s, *n* = 4.0,  *E* = 470000 J/mol, *V* = 8 J/mol/MPa,  σ_cr_ = 3 × 10^7^ Pa, C = 1 MPa, φ = 0 .05, μ = 67 GPa |

**Table S2. Conditions and results of numerical experiments**

| **Model**  **Resolution** | **Crust:**  **upper, km**  **middle, km**  **lower, km** | **S-India**  **L, km**  **H, km** | **N-India**  **L, km**  **H, km** | **Tibet**  **L, km**  **H, km** | **Tarim**  **L, km**  **H, km** | **Tien-Shan**  **L, km**  **H, km** | **Kazakh Plate**  **L, km**  **H, km** | **Results** |
| --- | --- | --- | --- | --- | --- | --- | --- | --- |
| ivad  601x  151 | 20  20  0 | 700  100 | 1050  200 | 1400  70 | 700  200 | 1900  80-100-80^a^ | 250  150 | At 0 Ma lithospheric shortening starts in at the S-boundary of Tibet and propagates to the North, at 17 Ma shortening starts at the S-boundary of Tien-Shan and propagates to the North, the experiment stopped at 44 Ma before the onset of shortening in Central India. |
| ivae  601x  151 | 20  20  0 | 700  100 | 1050  200 | 1400  75 | 700  200 | 1900  80-100-80^a^ | 250  150 | At 0 Ma lithospheric shortening starts in at the S-boundary of Tibet and propagates to the North, at 16 Ma shortening starts at the S-boundary of Tien-Shan and propagates to the North, the experiment stopped at 43 Ma before the onset of shortening in Central India. |
| ivaf  601x  151 | 25  0  15 | 700  100 | 1050  200 | 1400  80 | 700  200 | 1900  80-100-80^a^ | 250  150 | At 0 Ma lithospheric shortening starts in at the S-boundary of Tibet and propagates to the North, at 10 Ma shortening starts at the S-boundary of Tien-Shan and propagates to the North, at 17 Ma shortening starts at the N-boundary of Tien-Shan and propagates to the South, the experiment stopped at 46 Ma before the onset of shortening in Central India. |
| ivag  601x  151 | 15  10  15 | 700  100 | 1050  200 | 1400  80 | 700  200 | 1900  80-100-80^a^ | 250  150 | At 0 Ma lithospheric shortening starts in at the S-boundary of Tibet and propagates to the North, at 10 Ma shortening starts at the S-boundary of Tien-Shan and propagates to the North, at 15 Ma shortening starts at the N-boundary of Tien-Shan and propagates to the South, at 42 Ma shortening starts in Central India. |
| ivah  601x  151 | 15  10  15 | 700  100 | 1050  200 | 1400  90 | 700  200 | 1900  90-100-90^a^ | 250  150 | At 0 Ma lithospheric shortening starts in at the S-boundary of Tibet and propagates to the North, at 12 Ma shortening starts at the S-boundary of Tien-Shan and propagates to the North, at 15 Ma shortening starts at the N-boundary of Tien-Shan and propagates to the South, at 43 Ma shortening starts in Central India. |
| ivai  601x  151 | 15  15  10 | 700  100 | 1050  200 | 1400  90 | 700  200 | 1900  90-100-90^a^ | 250  150 | At 0 Ma lithospheric shortening starts in at the S-boundary of Tibet and propagates to the North, at 15 Ma shortening starts at both the N-boundary and the S-boundary of Tien-Shan and propagates to the middle of it, at 35 Ma shortening starts in Central India. |
| ivaj  1201x  151 | 15  15  10 | 700  100 | 1050  200 | 1400  90 | 700  200 | 1900  90-100-90^a^ | 250  150 | At 0 Ma lithospheric shortening starts in at the S-boundary of Tibet and propagates to the North, at 15 Ma shortening starts at both the N-boundary and the S-boundary of Tien-Shan and propagates to the middle of it, at 35 Ma shortening starts in Central India. |
| ivak  1201x  151 | 15  10  15 | 700  100 | 1050  200 | 1400  90 | 700  200 | 1900  90-100-90^a^ | 250  150 | At 0 Ma lithospheric shortening starts in at the S-boundary of Tibet and propagates to the North, at 12 Ma shortening starts at the S-boundary of Tien-Shan and propagates to the North, at 15 Ma shortening starts at the N-boundary of Tien-Shan and propagates to the South, at 43 Ma shortening starts in Central India. |
| ival  1201x  151 | 15  10  15 | 700  100 | 1050  200 | 1400  90 | 700  200 | 1900  95-100-95^a^ | 250  150 | At 0 Ma lithospheric shortening starts in at the S-boundary of Tibet and propagates to the North, at 20 Ma shortening starts at the N-boundary of Tien-Shan and propagates to the South, at 30 Ma shortening starts at the S-boundary of Tien-Shan, at 40 Ma shortening starts in Central India. |
| ivam  1201x  151 | 15  10  15 | 700  105 | 1050  200 | 1900  90 | 700  200 | 900  95 | 750  150 | At 0 Ma lithospheric shortening starts in at the S-boundary of Tibet and propagates to the North, at 20 Ma shortening starts at the N-boundary of Tien-Shan and propagates to the South, at 45 Ma shortening starts in Central India. |
| ivan  1201x  151 | 15  10  15 | 700  105 | 1050  200 | 1900  90 | 700  200 | 900  95-100 ^b^ | 750  150 | At 0 Ma lithospheric shortening starts in at the S-boundary of Tibet and propagates to the North, at 20 Ma shortening starts at the S-boundary of Tien-Shan and propagates to the North, at 45 Ma shortening starts in Central India. |
| ivao^c^  1201x  151 | 15  10  15 | 700  105 | 1050  200 | 1900  90-95^b^ | 700  200 | 900  95-100 ^b^ | 750  150 | At 0 Ma lithospheric shortening starts in at the S-boundary of Tibet and propagates to the North, at 20 Ma shortening starts at the S-boundary of Tien-Shan and propagates to the North, at 45 Ma shortening starts in Central India. |

**Table S2. Conditions and results of numerical experiments**

^a^ lithospheric thickness first increases and then decreases from South to North

^b^ lithospheric thickness increases from South to North

^c^ reference model
